# Sensory Processing and Integration at the Carotid Body Tripartite Synapse: Neurotransmitter Functions and Effects of Chronic Hypoxia

**DOI:** 10.3389/fphys.2018.00225

**Published:** 2018-03-16

**Authors:** Erin M. Leonard, Shaima Salman, Colin A. Nurse

**Affiliations:** Department of Biology, McMaster University, Hamilton, ON, Canada

**Keywords:** carotid body, chemoreceptor type I cells, glial-like type II cells, purinergic signaling, neurotransmitters, sensory transmission, petrosal neurons

## Abstract

Maintenance of homeostasis in the respiratory and cardiovascular systems depends on reflexes that are initiated at specialized peripheral chemoreceptors that sense changes in the chemical composition of arterial blood. In mammals, the bilaterally-paired carotid bodies (CBs) are the main peripheral chemoreceptor organs that are richly vascularized and are strategically located at the carotid bifurcation. The CBs contribute to the maintenance of O_2_, CO_2_/H^+^, and glucose homeostasis and have attracted much clinical interest because hyperactivity in these organs is associated with several pathophysiological conditions including sleep apnea, obstructive lung disease, heart failure, hypertension, and diabetes. In response to a decrease in O_2_ availability (hypoxia) and elevated CO_2_/H^+^ (acid hypercapnia), CB receptor type I (glomus) cells depolarize and release neurotransmitters that stimulate apposed chemoafferent nerve fibers. The central projections of those fibers in turn activate cardiorespiratory centers in the brainstem, leading to an increase in ventilation and sympathetic drive that helps restore blood PO_2_ and protect vital organs, e.g., the brain. Significant progress has been made in understanding how neurochemicals released from type I cells such as ATP, adenosine, dopamine, 5-HT, ACh, and angiotensin II help shape the CB afferent discharge during both normal and pathophysiological conditions. However, type I cells typically occur in clusters and in addition to their sensory innervation are ensheathed by the processes of neighboring glial-like, sustentacular type II cells. This morphological arrangement is reminiscent of a “tripartite synapse” and emerging evidence suggests that paracrine stimulation of type II cells by a variety of CB neurochemicals may trigger the release of “gliotransmitters” such as ATP via pannexin-1 channels. Further, recent data suggest novel mechanisms by which dopamine, acting via D2 receptors (D2R), may inhibit action potential firing at petrosal nerve endings. This review will update current ideas concerning the presynaptic and postsynaptic mechanisms that underlie chemosensory processing in the CB. Paracrine signaling pathways will be highlighted, and particularly those that allow the glial-like type II cells to participate in the integrated sensory response during exposures to chemostimuli, including acute and chronic hypoxia.

## Introduction

Oxygen (O_2_) is essential to the survival of aerobic organisms that rely on oxidative phosphorylation for the production of ATP as a major energy source. Consequently, both water- and air-breathing vertebrates have evolved mechanisms for monitoring O_2_ levels in the environmental water, pulmonary airways, and/or arterial blood. In conditions of O_2_ deficiency (hypoxia), strategically-located sensors in the gills of water-breathers and peripheral chemoreceptor organs of air-breathers initiate compensatory respiratory and cardiovascular reflex responses so as to maintain homeostasis (Gonzalez et al., 1994; Cutz and Jackson, 1999; Milsom and Burleson, 2007; Kumar and Prabhakar, 2012). In mammals, the main peripheral chemoreceptor organs are the carotid bodies (CBs), which contain O_2_- and CO_2_/H^+^-sensitive detectors known as glomus or type I cells (Gonzalez et al., 1994; Peers and Buckler, 1995; López-Barneo et al., 2016). While the chemoreceptive properties of the CB have been well established over much of the last century (Gonzalez et al., 2014), current views consider the CBs as general metabolic sensors capable of detecting not only the respiratory gases and blood acidity, but also blood glucose and circulating insulin levels (López-Barneo, 2003; Conde et al., 2014; Thompson et al., 2016).

The CBs are located bilaterally at the carotid bifurcation where they monitor blood O_2_ just before it reaches the brain, an organ that is a critically sensitive to O_2_ deprivation. In keeping with their role as circulatory chemical sensors, they are reputed to be the most richly vascularized organs in the body (Gonzalez et al., 1994). The gross morphology of the CB reveals a network of fenestrated capillaries that penetrate clusters of chemoreceptor type I cells which receive sensory innervation from the carotid sinus nerve (CSN) (McDonald, 1981). The type I cells share an intimate association with neighboring glial-like, sustentacular type II cells in a ratio of ~4:1 (McDonald, 1981; Kondo, 2002), and there is evidence of synapse-like specializations between these two cell types (Platero-Luengo et al., 2014). Thus, the CB chemoreceptor complex, consisting of clusters of receptor type I cells, type II glial cells, and the abutting sensory nerve terminals, displays the features of a “tripartite synapse” that provides the substrate for synaptic integration at many sites in the central nervous system (CNS) (Eroglu and Barres, 2010). During chemoexcitation, the CB output is relayed as an increase in action potential frequency in chemosensory fibers of the CSN whose cell bodies are located in the petrosal ganglia. These fibers terminate centrally in the nucleus tractus solitarius where they help control lung ventilation and sympathetic outflow to the cardiovascular system (Claps and Torrealba, 1988; Gonzalez et al., 1994; Kumar and Prabhakar, 2012).

Over the last ~30 years there have been numerous studies on the transduction mechanisms by which CB receptor type I cells sense a reduction in PO_2_ (hypoxia) and an elevation in PCO_2_/H^+^ and how these signals are relayed to the brain via the CSN. On the presynaptic side, there is a consensus that acute hypoxia causes inhibition of background K^+^ channels in type I cells, and this is a key step in chemotransduction leading to voltage-gated Ca^2+^ entry and neurotransmitter release (Buckler, 2015; López-Barneo et al., 2016). However, despite significant recent progress, the identity of the PO_2_ sensor is still debatable though there is strong support for a role of the mitochondrial electron transport chain (Buckler, 2015; López-Barneo et al., 2016; Chang, 2017; Prabhakar and Peng, 2017; Rakoczy and Wyatt, 2017). On the postsynaptic side, there has been significant progress in elucidating the roles of several neurochemicals, especially the purines ATP and its metabolite adenosine, in shaping the afferent CSN discharge (Iturriaga and Alcayaga, 2004; Nurse, 2005, 2010; Conde et al., 2012a; Nurse and Piskuric, 2013). However, this task has become more challenging given the increasing number of neuroactive agents present in the CB, and the growing evidence that the glial-like type II cells may not be silent partners during sensory transmission (Tse et al., 2012; Nurse and Piskuric, 2013; Nurse, 2014). An understanding of the roles of these neurochemicals and the mechanisms underlying synapse integration in the CB is important given that alterations in CB sensitivity and CSN discharge are now linked to several cardiorespiratory abnormalities in humans (Kumar and Prabhakar, 2012; Iturriaga, 2017). For example during obstructive sleep apnea, a condition where patients are exposed to bouts of chronic intermittent hypoxia (CIH), CB chemosensitivity becomes exaggerated leading to long-term facilitation (LTF) of the sensory discharge and increased risk of hypertension (Prabhakar et al., 2015). Also, CB chemosensitivity is enhanced in chronic heart failure (CHF), leading to sympathetic hyperactivity and exacerbation of the disease progression (Schultz et al., 2015). In this article, we review the role of several neurotransmitters and neuromodulators in the integrative sensory response at the CB tripartite synapse. In particular, we consider the major transmitters involved in the interactions between type I cells and the sensory nerve ending during chemotransduction, as well as the contribution of paracrine signaling mechanisms to synaptic integration and crosstalk among type I cells, type II cells, and the sensory nerve endings. Finally, we consider how synaptic plasticity mechanisms during exposures to chronic sustained hypoxia might contribute to alterations in neurochemical signaling at the tripartite synapse.

## Synaptic transmission between chemoreceptor type I cells and sensory nerve terminals

During chemotransduction type I cells release neurotransmitters resulting in an increase in CSN action potential frequency. As discussed below, the main transmitters responsible for this postsynaptic excitatory response are likely ATP and adenosine; however, contributions from other CB transmitters including ACh, histamine, and 5-HT cannot be ignored and may well depend on developmental age, species under investigation, or the presence or absence of pathophysiological conditions (Zhong et al., 1999; Iturriaga and Alcayaga, 2004; Nurse, 2005, 2010; Lazarov et al., 2006; Del Rio et al., 2008; Conde et al., 2012a; Kumar and Prabhakar, 2012). Further, the magnitude of the CSN excitatory response appears to be blunted by the concurrent action of inhibitory neurotransmitters such as GABA and dopamine (Alcayaga et al., 1999; Iturriaga et al., 2009; Nurse, 2010). In the case of dopamine, it is possible that its release from activated afferent C fibers during chemoexcitation may lead to autocrine-paracrine stimulation of inhibitory D2 receptors (D2R) on nearby type I cells and/or afferent nerve terminals (Benot and Lopez-Barneo, 1990; Almaraz et al., 1993; Iturriaga et al., 2003).

### Role of ATP and postsynaptic P2X2/3 receptors

**Figure 1:**
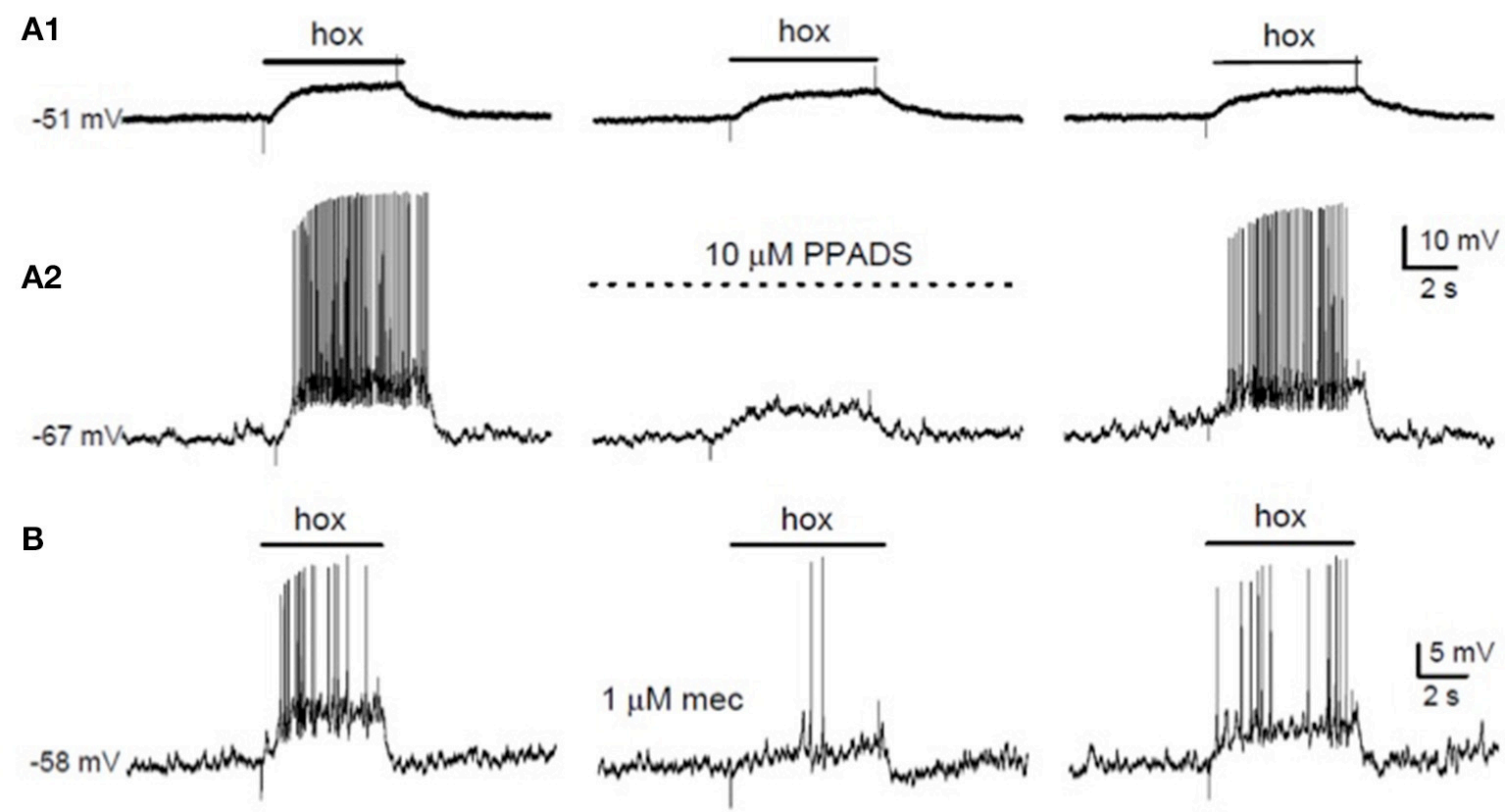
Effects of purinergic and nicotinic receptor blockers on hypoxic chemotransmission in co-cultures. **(A)** Simultaneous recordings of membrane potential from a type I cell **(A1)** and a petrosal neuron **(A2)** in co-culture. The P2X2/3 receptor blocker PPADS (10 μM) reversibly suppressed the “postsynaptic” neuronal response **(A2)**, but had negligible effect on the “presynaptic” type I cell response **(A1)**. **(B)** The hypoxia (hox)-induced sensory discharge in a co-cultured petrosal neuron (left trace) was reversibly inhibited by the nicotinic cholinergic blocker mecamylamine (mec; 1 μM; middle trace). Data adapted from Nurse (2010).

The stimulatory effects of intracarotid administration of ATP on ventilation and CSN discharge have been known for some time (reviewed by Zapata, 2007). However, use of a co-culture model of rat type I cell clusters and juxtaposed petrosal neurons demonstrated that blockers of purinergic P2X2/3 receptors, i.e., suramin and PPADS, inhibited the postsynaptic response elicited by hypoxia and/or hypercapnia (Zhang et al., 2000; Prasad et al., 2001; Zhang and Nurse, 2004; Nurse and Piskuric, 2013). A central role of ATP acting via postsynaptic P2X2/3 receptors during chemoexcitation was confirmed by the following observations: (i) Chemosensitive petrosal neurons in co-culture expressed functional P2X2/3 receptors (Zhang et al., 2000); (ii) Immunoreactive P2X2 and P2X3 subunits were localized to petrosal terminals opposed to type I cells in CB tissue sections *in situ* (Zhang et al., 2000; Prasad et al., 2001); (iii) P2X2 knockout mice showed a markedly attenuated hypoxic ventilatory response (Rong et al., 2003); (iv) Acute hypoxia induced Ca^2+^-dependent, vesicular ATP release from isolated whole CB, CB slices, and cultured CB cells (Buttigieg and Nurse, 2004; Conde et al., 2012a); (v) In intact CB-sinus nerve preparations *in vitro*, P2X2/3 and selective P2X3 receptor antagonists inhibited the CSN discharge evoked by hypoxia (He et al., 2006; Reyes et al., 2007; Niane et al., 2011); and (vi) In the co-culture model, the selective P2X receptor blocker PPADS inhibited the hypoxia-induced postsynaptic response in the petrosal neuron, but not the presynaptic receptor potential in type I cells (Figures 1A1,A2). Though most of the preceding data were obtained in rodent preparations, an excitatory postsynaptic role of ATP acting via ligand-gated receptor channels has also been observed in the cat CB (Iturriaga and Alcayaga, 2004; Reyes et al., 2007; Zapata, 2007).

### Role of adenosine and postsynaptic A2a receptors

The excitatory effects of exogenous adenosine on both ventilation and CSN discharge were first confirmed in the 1980's and appear to be species independent (McQueen and Ribeiro, 1983; Monteiro and Ribeiro, 1987; Conde et al., 2006, 2009). While presynaptic adenosine A2a and A2b receptors on type I cells are well described (see later), the excitatory effects of exogenous adenosine appear to be mainly postsynaptic, involving high affinity A2a receptors (A2aR) that are expressed in petrosal chemoafferent neurons (Gauda, 2002; Conde et al., 2006, 2009). During acute hypoxia and hypercapnia a significant fraction of the CSN sensory discharge is dependent on the stimulation of postsynaptic A2aR (Conde et al., 2006; Zhang et al., 2017). The extracellular adenosine level at the CB chemosensory synapse is generated via equilibrative nucleoside transporters (ENTs) in type I cells and/or breakdown of extracellular ATP by ectonucleotidases (Conde and Monteiro, 2004). More recently, molecular characterization and immunolocalization of surface-located nucleotidases in the rat CB have revealed the presence of nucleotidase triphosphate diphospho-hydrolase (NTPDase)2,3 and ecto-5′nucleotidase(ecto-5′Nt/CD73) in association with type I cell clusters (Salman et al., 2017). The NTPDase 2,3 pair efficiently hydrolyses ATP to AMP and ADP, while ecto-5′Nt hydrolyses AMP to adenosine (Zimmermann, 2000). Interestingly, in a recent study pharmacological inhibition of ecto-5′NT, but not ENTs, caused a reduction in basal and hypoxia-evoked CB sensory discharge, as well as in the hypoxic ventilatory response in adult rats (Holmes et al., 2017). These data suggest that the principal source of adenosine that contributes to the increase in CSN discharge during acute hypoxia is from the catabolism of extracellular ATP. However, in a previous study, pharmacological inhibition of ENT was found to increase adenosine release from rat CBs especially at high-intensity hypoxia, at least when adenosine deaminase was simultaneously inhibited (Conde et al., 2012a). Thus, the relative contributions of ecto-5′Nt and ENT to extracellular adenosine during acute hypoxia may well depend on the PO_2_ level. A plausible mechanism by which adenosine, acting via postsynaptic A2aR, increases excitability in petrosal afferent neurons was recently proposed (Zhang et al., 2017). In that study on rat CB co-cultures, adenosine caused a depolarizing shift in the voltage dependence of activation of a hyperpolarization-activated cyclic nucleotide gated cation current *I*h in chemosensory neurons. Consistent with the involvement of high affinity A2aR, the effect was mediated by nanomolar concentrations of adenosine and was reversibly inhibited by the selective A2aR blocker, SCH58261. In concert with the known effects of the *I*h current in regulating firing frequency in a variety of cell types (Biel et al., 2009), adenosine was also shown to increase membrane excitability and action potential frequency in identified chemosensory petrosal neurons in co-culture (Zhang et al., 2017). Moreover, HCN4 immunoreactive subunits were localized to chemosensory, tyrosine hydroxylase (TH)-positive petrosal neurons in tissue sections of rat petrosal ganglia *in situ* (Zhang et al., 2017); HCN4 subunits are known to contribute to *I*h channel currents and to increases in action potential frequency through elevations in intracellular cAMP in different cell types (Biel et al., 2009).

### Role of ACh and nicotinic receptors

The role of ACh as a major excitatory neurotransmitter in the CB has had a controversial history (Eyzaguirre and Zapata, 1984; Nurse and Zhang, 1999; Fitzgerald, 2000; Iturriaga and Alcayaga, 2004). There is no doubt that various nicotinic and muscarinic ACh receptors (AChR) are expressed in the CB of several species, though whether or not type I cells synthesize and store ACh in all cases is controversial (Gauda, 2002; Iturriaga and Alcayaga, 2004; Zhang and Nurse, 2004; Shirahata et al., 2007). However, hypoxia-induced release of ACh from intact CBs has been detected in several species including cats, rabbits, and humans (Fitzgerald, 2000; Shirahata et al., 2007; Zapata, 2007; Kåhlin et al., 2014). Moreover, in the co-culture model of the rat CB a combination of nicotinic and purinergic blockers were required to inhibit most of the hypoxia-induced postsynaptic response (Zhang et al., 2000); often, only partial inhibition was seen when either blocker was present alone (Figures 1A2,B). In concert with these results, systemic blockade of both nicotinic and purinergic P2X receptors inhibited ventilation in newborn rats (Niane et al., 2012). Additional evidence supporting ACh as an important CB excitatory neurotransmitter was obtained in the following studies: (i) functional nicotinic AChR were present on >65% of isolated rat petrosal neurons (Zhong and Nurse, 1997) and in the majority of petrosal neurons that were functionally connected to the cat CB (Varas et al., 2003; Iturriaga and Alcayaga, 2004); (ii) in the isolated rat and cat CB-sinus nerve preparations *in vitro* nicotinic AChR blockers inhibited the hypoxia-induced chemosensory discharge (Iturriaga and Alcayaga, 2004; He et al., 2005; Zapata, 2007; Niane et al., 2009); and (iii) consistent with a postsynaptic role for ACh, α7 nAChR immunoreactivity has been localized to nerve endings surrounding type I clusters in rat and cat CB *in situ* (Shirahata et al., 2007; Niane et al., 2009), and there is electrophysiological evidence consistent with the presence of functional α7 nAChR in cat petrosal neurons (Alcayaga et al., 2007).

### Role of histamine and H1-3 receptors

Histamine is synthesized, stored (in considerably higher amounts than dopamine), and released from the CB during acute hypoxia (Koerner et al., 2004; Del Rio et al., 2008). In addition, histamine receptors (H1, H2, H3) have been localized to both the CB and petrosal ganglion (Lazarov et al., 2006). Application of H1R and H3R agonists to the rat CB had a mild stimulatory effect on ventilatory output (Lazarov et al., 2006). In the cat, H1R antagonists reduced, whereas H3R antagonists enhanced, the excitatory effect of histamine on the sinus nerve discharge (Del Rio et al., 2008). In the latter study, though histamine increased sinus nerve discharge when applied to both the isolated superfused and perfused CB *in vitro*, it had no effect on the isolated petrosal ganglion discharge. This suggests a direct effect of histamine on the CB parenchymal cells and/or a selective effect on petrosal terminals that is not replicated at the soma. In isolated rat type I cells, H3R agonists inhibited the rise in Ca^2+^ elicited by muscarinic agonists (Thompson et al., 2010). Further work is required to clarify the role of histamine and its receptors in the CB though both excitatory and inhibitory pathways appear to be present.

### Role of inhibitory neurotransmitters

The preceding sections have focused on postsynaptic interactions that are predominantly excitatory. However, inhibitory mechanisms appear to play an important role in regulating the CB sensory discharge. Dopamine is probably the best-described inhibitory CB neurotransmitter (Benot and Lopez-Barneo, 1990), yet it was only recently that a plausible postsynaptic mechanism was proposed (Zhang et al., 2017). In the latter study on rat CB co-cultures, dopamine caused a hyperpolarizing shift in the voltage dependence of *I*_h_ activation and a decrease in membrane excitability in chemosensory petrosal neurons. This effect was opposite to that discussed above for adenosine, where the voltage dependence of *I*_h_ activation was shifted in the depolarizing direction and resulted in increased membrane excitability. The effect of dopamine on *I*_h_ was prevented during co-incubation with the selective D2 receptor (D2R) antagonist, sulpiride (Zhang et al., 2017). These data suggest that stimulation of D2R on chemosensory petrosal neurons and their terminals causes a decrease in intracellular cAMP leading to inhibition of the cAMP-gated *I*_h_ channels containing HCN4 subunits (Zhang et al., 2017). In this scenario, the source of dopamine is predominantly from nearby type I cells though it may include terminals of excited catecholaminergic C fibers of petrosal CB chemoafferents (Almaraz et al., 1993; Iturriaga et al., 2003).

There is also evidence that GABA, acting via ionotropic GABA(A) receptors on petrosal afferent terminals, can inhibit the chemosensory discharge by a shunting mechanism which diminishes the depolarizing action of excitatory inputs (Zhang et al., 2009). GABA is synthesized and stored in type I cells and its release during chemoexcitation has been inferred since pharmacological blockade of GABA(A) receptors using bicuculline facilitates the hypoxia-induced sensory discharge in cat and rat carotid body (Igarashi et al., 2009; Zhang et al., 2009). Moreover benzodiazepines, which enhance GABA(A) receptor activity, suppress ventilation in cats and inhibit the CB chemosensory discharge evoked by acute hypoxia (Igarashi et al., 2009).

## Autocrine-paracrine interactions involving chemoreceptor type I cells and glial-like type II cells

As discussed above, purinergic transmission from CB type I cells to the sensory nerve endings involves fast-acting ionotropic P2X receptors and slow-acting metabotropic adenosine P1 receptors. However, both autocrine and paracrine signaling mechanisms mediated mainly via metabotropic receptors on type I and glial-like type II cells can further fine-tune the synaptic neurotransmitter pool near the sensory nerve endings. In addition to direct synaptic interactions between type I cells and sensory nerve terminal the following cell- cell interactions are possible: (i) type I cell to type I cell; (ii) type I cell to type II cell; (iii) type II cell to type I cell; and (iv) direct communication between type II cells and the sensory nerve ending. Electrical coupling between adjacent type I cells, and between type I cells and the sensory nerve ending, is also possible as reviewed elsewhere (Eyzaguirre, 2007).

### Autocrine-paracrine signaling among neighboring type I cells

These pathways may involve either negative or positive feedback mechanisms. Perhaps the best-studied pathway involves the negative feedback role of dopamine acting on inhibitory D2R on the same or neighboring type I cells. For example, selective blockade of D2R using domperidone or haloperidol caused a concentration dependent enhancement of basal, high K^+^-, and hypoxia-evoked catecholamine release from intact rat CB (Conde et al., 2008). The effect is likely due to a negative feedback inhibition of intracellular Ca^2+^ signaling because dopamine was found to inhibit L-type Ca^2+^ currents in isolated type I cells (Benot and Lopez-Barneo, 1990), whereas selective D2R agonists inhibited the hypoxia-evoked rise in intracellular Ca^2+^ (Carroll et al., 2005). In addition to DA, there is evidence that ATP and GABA may also inhibit type I cell function via negative feedback mechanisms. For example, ATP was found to inhibit the hypoxia-induced rise in intracellular Ca^2+^ in type I cells via metabotropic P2Y1R (Xu et al., 2005) or P2Y12R (Carroll et al., 2012). Because ADP is also an effective ligand for both these receptors, and P2Y12Rs are highly expressed in type I cells (Zhou et al., 2016), it is possible that degradation of ATP by the surface-located nucleotidases NTPDase2,3 (Salman et al., 2017), generates sufficient ADP to further inhibit type I cells. Under normoxic conditions, NTPDase2 mRNA expression in the CB is much higher than NTPDase3 mRNA (Salman et al., 2017). Assuming parallel changes in the corresponding proteins, this expression pattern favors elevated ADP levels since NTPDase2 is relatively ineffective at hydrolyzing ADP to AMP (Zimmermann, 2000). GABA acting on autocrine-paracrine GABA(B) receptors was reported to inhibit the hypoxia-evoked receptor potential in type I cells (Fearon et al., 2003). In the latter study, the signaling mechanism involved activation of a resting TASK-like K^+^ conductance via a pertussis toxin- sensitive and PKA-dependent pathway.

The type I cell response to chemostimuli such as acute hypoxia may also be modified by positive feedback mechanisms. For example, the ATP metabolite adenosine has been shown to enhance intracellular Ca^2+^ signaling in type I cells by triggering membrane depolarization and voltage-gated Ca^2+^ entry via adenylate cyclase-PKA dependent inhibition of background TASK channels (Xu et al., 2006). Also, there is evidence that 5-HT acting via 5-HT2a receptors (5-HT2aR) can enhance the hypoxia-evoked receptor potential in type I cells via an autocrine-paracrine positive feedback mechanism involving PKC-mediated inhibition of resting and Ca^2+^-dependent K^+^ conductances (Zhang et al., 2003).

### ATP release from type 1 cells during chemostimulation can lead to intracellular Ca^2+^ elevations in nearby type II cells

**Figure 2:**
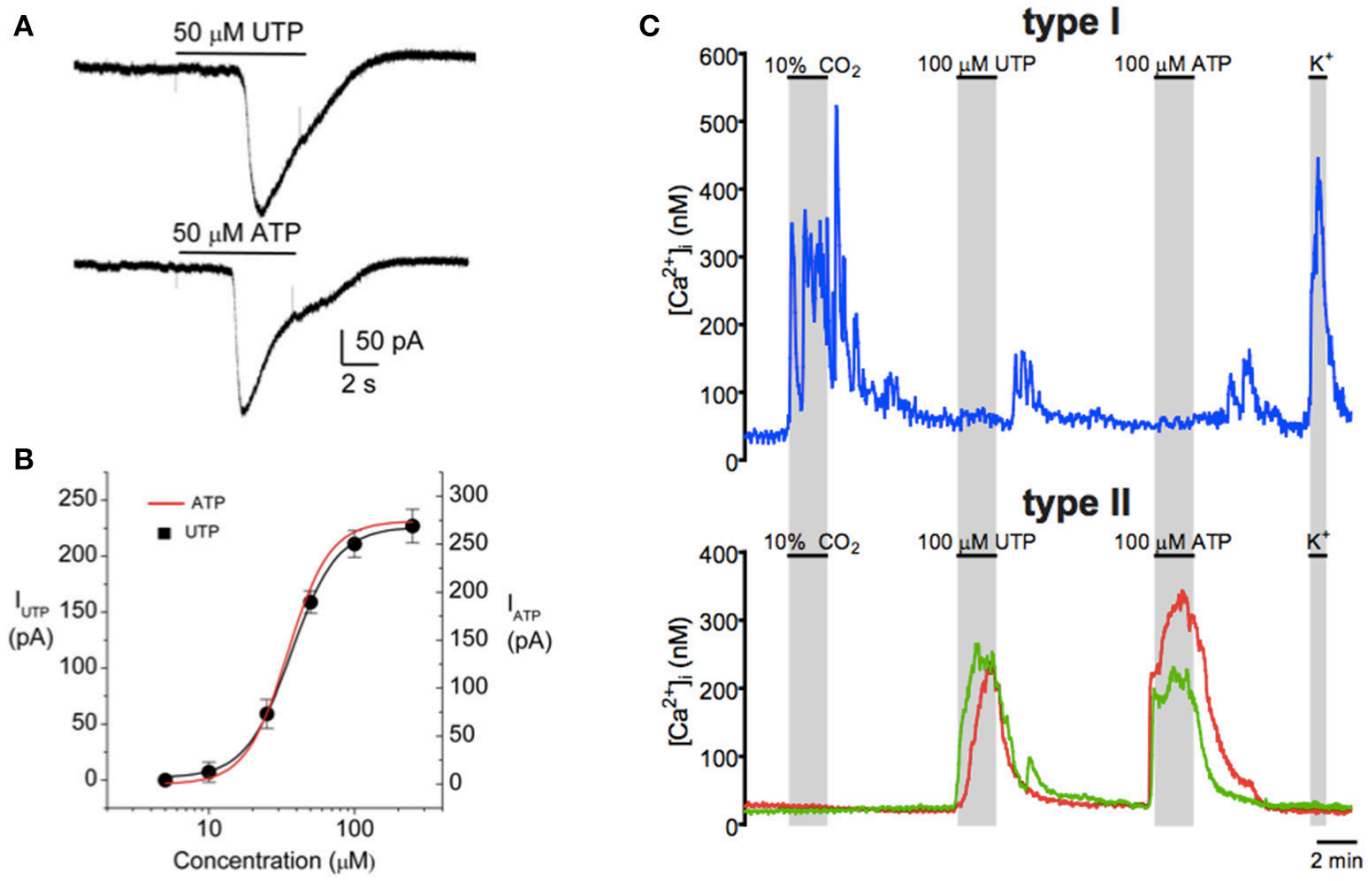
Purinergic activation of the inward current in type II cells is mediated by P2Y2 receptors in association with a rise in intracellular Ca^2+^. Both **(A)** ATP (50 μM) and **(B)** UTP (50 μM) induced similar inward currents (at −60 mV) in the same type II cell as expected for P2Y2 receptors. **(B)** Dose–response relation (black curve) for the UTP-evoked currents in a group of type II cells; data are represented as means ± SEM (*n* = 4) and fitted with the Hill equation with an EC_50_ = 37 μM. The dose–response curve for ATP (red) is superimposed and is indistinguishable from that for UTP. **(C)** Intracellular Ca^2+^ [Ca^2+^]_i_ responses monitored simultaneously in type I and type II cells. Both hypercapnia (10% CO_2_) and high K^+^ (30 mM) induced [Ca^2+^]_i_ responses in the type I cell (upper trace), but not in the two type II cells (lower red and green traces). Conversely, ATP and UTP induced similar [Ca^2+^]_i_ responses in the type II cells, but the type I cell was unresponsive. Data taken from Zhang et al. (2012).

**Figure 3:**
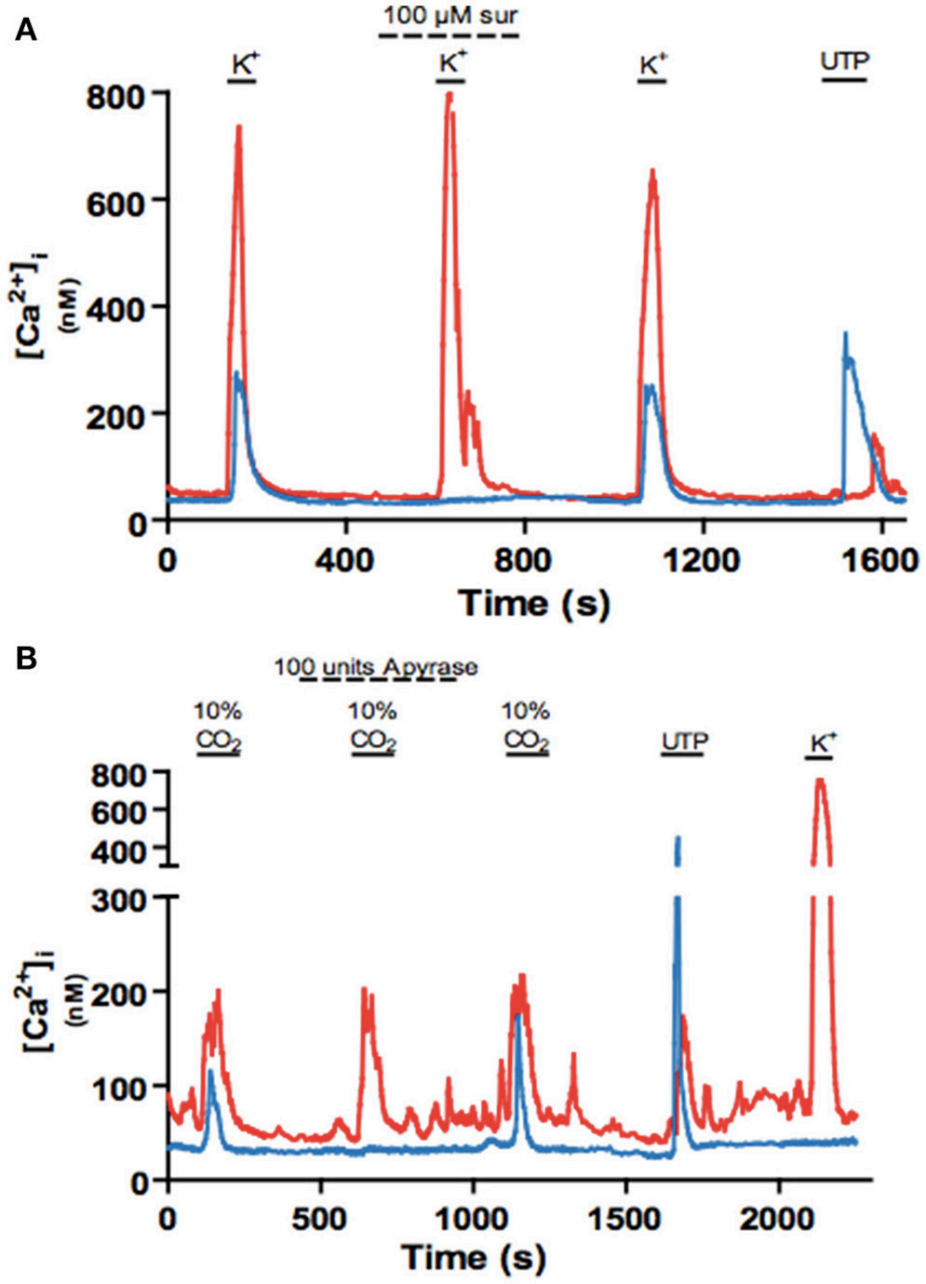
Blockade of P2Y2 receptors with suramin or degradation of extracellular ATP with apyrase inhibits crosstalk from type I to type II cells. **(A)** Example intracellular Ca^2+^ traces showing the reversible inhibition of the delayed or indirect Ca^2+^ response in a type II cell (blue) by the P2Y2R blocker suramin (100 μM) during stimulation of nearby type I cells (red) with high K^+^ (30 mM). **(B)** Application of apyrase (a nucleoside hydrolase) reversibly inhibits the delayed Ca^2+^ response in a type II cell during stimulation of nearby type I cells with high (10%) CO_2_. Data adapted from Murali and Nurse (2016).

The idea that glial-like type II cells may participate in the integrative CB sensory response was first suggested by the observation that the excitatory transmitter ATP can elicit a rise in intracellular Ca^2+^ in isolated type II cells (Xu et al., 2003). Though these cells display small voltage-dependent outward currents, they lack significant inward Na^+^ or Ca^2+^ currents (Duchen et al., 1988; Xu et al., 2003; Zhang et al., 2012), consistent with an intracellular source for the ATP-mediated Ca^2+^ rise (Xu et al., 2003). This effect of ATP was mimicked by selective P2Y2 receptor (P2Y2R) agonists such as UTP, and P2Y2R immunoreactivity was detectable in spindle-shaped type II cells in tissue sections of the rat CB *in situ* (Xu et al., 2003). As exemplified in Figure 2C, subsequent studies using the agonist UTP confirmed the P2Y2R-mediated increase in intracellular Ca^2+^ in rat type II cells (Piskuric and Nurse, 2012; Zhang et al., 2012). P2Y2Rs typically couple via G_q_-proteins to activate the phospholipase C-IP3-PKC signaling pathway and mobilize Ca^2+^ from internal stores (von Kügelgen, 2006), suggesting this mechanism is likely responsible for the Ca^2+^ elevation in type II cells. Because ATP is a key excitatory CB neurotransmitter the question arose whether paracrine stimulation of type II cells by ATP occurs during normal CB chemoexcitation. To address this, isolated rat type I cell clusters containing contiguous type II cells where challenged with chemosensory stimuli such as acute hypoxia and hypercapnia, as well as the depolarizing stimulus, high (30 mM) K^+^ (Murali and Nurse, 2016). As expected, all three stimuli evoked rapid intracellular Ca^2+^ elevations in receptor type I cells but, interestingly, nearby type II cells frequently responded with a delayed, secondary increase in intracellular Ca^2+^ (Murali and Nurse, 2016), as illustrated in Figures 3A,B. The paracrine action of ATP released from type I cells appeared to be responsible for the secondary type II cell Ca^2+^ responses because the latter were reversibly inhibited by the P2Y2R antagonist, suramin (Figure 3A), as well as by the nucleoside hydrolase, apyrase (Figure 3B).

### Several other CB neurochemicals stimulate a rise in intracellular Ca^2+^ in type II cells

In addition to ATP (see above), several other neuroactive chemicals in the CB cause a rise in intracellular Ca^2+^ when applied to a significant proportion of isolated type II cells. For example, the small molecule neurotransmitters ACh and 5-HT, acting via metabotropic muscarinic and 5-HT2 receptors respectively, stimulated intracellular Ca^2+^ transients in ~53 and 67% of UTP-sensitive type II cells respectively (Tse et al., 2012; Murali et al., 2015, 2017). In addition, nanomolar concentrations of the neuropeptide angiotensin II stimulated a rise in intracellular Ca^2+^ in a significant proportion (~75%) of type II cells via losartan-sensitive AT_1_ receptors (Tse et al., 2012; Murali et al., 2014). The biosynthetic pathway for producing angiotensin II, including angiotensin converting enzyme (ACE) and the precursor angiotensinogen, is expressed in type I cells (Leung et al., 2000; Lam and Leung, 2003), suggesting it could play a paracrine role in the CB. In all cases, the intracellular Ca^2+^ response in type II cells persisted in Ca^2+^-free extracellular solutions and/or following store depletion with thapsigargin or cyclopiazonic acid, suggesting Ca^2+^ release from internal stores (Tse et al., 2012; Murali et al., 2014, 2017). Although less well-studied, another neuropeptide, i.e., endothelin-1 (ET-1), that is synthesized and released by type I cells during hypoxia (Chen et al., 2002; Platero-Luengo et al., 2014), is also capable of evoking intracellular Ca^2+^ responses in type II cells at nanomolar concentrations (Murali et al., 2015). Whether or not all of these neuroactive agents combine to enhance Ca^2+^ signaling in type II cells during chemoexcitation, as described above for ATP, remains to be determined. It is also possible that their actions may become more relevant in pathophysiological conditions associated with chronic or intermittent hypoxia, when their expression level and/or that of their cognate receptors are elevated (Chen et al., 2002; Lam et al., 2004).

## Downstream effects of neurotransmitter-mediated intracellular Ca^2+^ signaling in type II cells

There is abundant evidence that glial cells in the CNS contribute to synapse integration by generating intracellular Ca^2+^ signals and releasing gliotransmitters such as ATP, GABA, and glutamate (Eroglu and Barres, 2010; Bazargani and Attwell, 2016). As discussed above, several CB neurotransmitters elicited intracellular Ca^2+^ elevations in type II cells raising questions about the downstream consequence, and particularly whether this led to the release of gliotransmitters.

### Carotid body neurotransmitters activate Pannexin-1 (Panx-1) channels in type II cells

**Figure 4:**
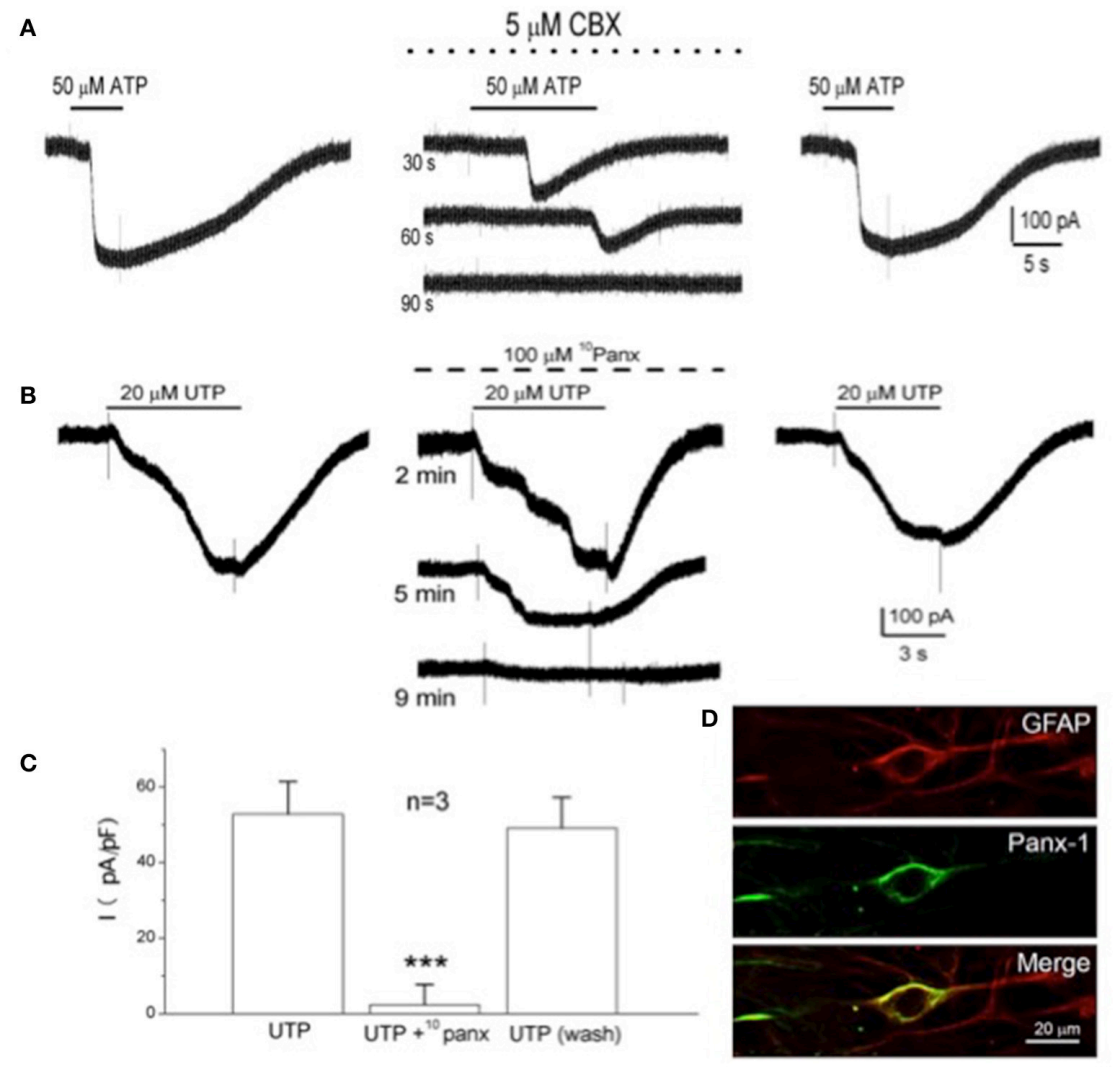
Inhibition of the P2Y2R-activated inward current by selective Panx-1 channel blockers and localization of Panx-1 immunoreactivity in type II cells. The selective Panx-1 blockers, carbenoxolone (CBX; 5 μM) and ^10^Panx peptide (100 μM), reversibly inhibited the inward current in type II cells elicited by 50 μM ATP **(A)** and 20 μM UTP **(B)**, respectively. **(C)** UTP-activated inward current (plotted as current density pA/pF) for three cells before, during and after (wash) treatment with 100 μM ^10^Panx peptide; ^***^P < 0.001. **(D)** Confocal images of pannexin-1 (Panx-1) immunostaining of a solitary, GFAP-positive, spindle-shaped type II cell in a 6-day-old culture of dissociated rat carotid body; note co-localization of GFAP-ir with Panx-1-ir in the type II cell. Adapted from Zhang et al. (2012) and Murali et al. (2017).

In electrophysiological studies on isolated type II cells, the P2Y2R agonists ATP or UTP activated an inward current at the resting membrane potential (Zhang et al., 2012), as illustrated in Figures 2A,B. This current reversed direction near 0 mV and, as illustrated in Figure 4A, was reversibly inhibited by low concentrations of carbenoxolone (5 μM), a putative blocker of pannexin-1 (Panx-1) channels. Panx-1 immunoreactivity also co-localized with isolated GFAP-positive type II cells in dissociated CB cultures (see Figure 4D), and stained processes of type II cells in CB tissue sections (Zhang et al., 2012). Panx-1 channels assemble as hexamers in the plasma membrane and though structurally similar to gap junction hemichannels, Panx-1 sequences show closer homology to the invertebrate innexins (Dahl, 2015; Penuela et al., 2015). In addition to ATP, other CB neurotransmitters that evoked a rise in intracellular Ca^2+^ in type II cells such as angiotensin II, ACh, and 5-HT also activated an inward Panx-1-like current based on its sensitivity to carbenoxolone (Murali et al., 2014, 2015, 2017). However, carbenoxolone is also a well-known blocker of gap junctional connexins, albeit at typically higher (>10x) concentrations (Lohman and Isakson, 2014), raising questions about the true identity of the channels. In recent studies, the UTP-evoked inward current in type II cells was reversibly inhibited by a more specific Panx-1 mimetic peptide channel blocker ^10^Panx (100 μM) (see Figures 4B,C), but not by similar concentrations of scrambled control peptide ^sc^Panx (Murali et al., 2017). Taken together, the combined pharmacological and immunocytochemical data, summarized in Figure 4, support the notion that Panx-1 channels mediate the neurotransmitter-activated inward currents in type II cells.

### Activation of Pannexin-1 channels in type II cells is dependent on intracellular Ca^2+^

**Figure 5:**
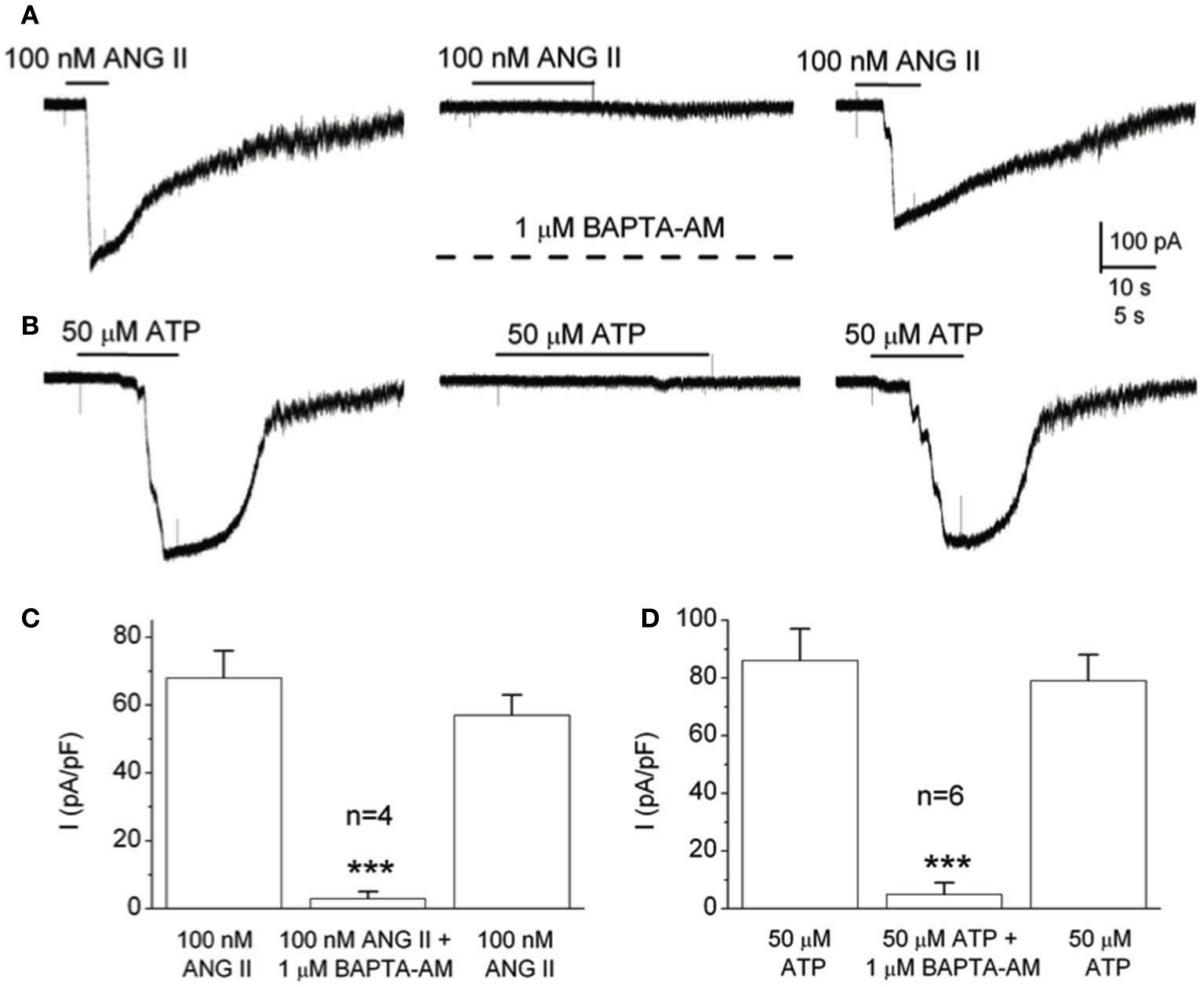
Chelating intracellular Ca^2+^ in type II cells with BAPTA prevents Panx-1 current activation by angiotensin II and ATP. **(A)** The ANG II-induced Panx-1 current was almost completely and reversibly inhibited during incubation with the membrane permeable Ca^2+^ chelator, BAPTA-AM (1 μM; middle trace). **(B)** Similarly, the same result was obtained when ATP was used as the agonist. **(C,D)** Summary data show the reversible inhibition of the Panx-1 current evoked by ANG II and ATP respectively, when BAPTA-AM was present (**C**, *n* = 4, and **D**, *n* = 6); ^***^p < 0.001. Data taken from Murali et al., 2014.

The observation that several neurotransmitters that elicited a rise in intracellular Ca^2+^ also activated Panx-1 currents in type II cells led to an investigation of a possible link between the two events (Murali et al., 2014). Indeed, as illustrated in Figures 5A–D, the Panx-1 inward current activated by either angiotensin II or ATP was reversibly inhibited when the membrane-permeable Ca^2+^ chelator BAPTA-AM was present in the extracellular solution (Murali et al., 2014). These results are in concert with previous demonstrations that: (i) ATP activated an inward current at negative potentials in oocytes co-expressing Panx-1 channels and P2Y2R (Locovei et al., 2006); (ii) in inside-out patches from these oocytes Panx-1 channels were strongly activated at negative potentials when the cytoplasmic face was exposed to micromolar (but not 0) Ca^2+^; and (iii) in other glial cell types, e.g., microglia and astrocytes, Panx-1 channel opening is regulated by intracellular Ca^2+^ (Dahl, 2015). However, it should be noted that the activation of Panx-1 channels by cytoplasmic Ca^2+^ may be dependent on cell type, since in hippocampal neurons Panx-1 channel activation is Ca^2+^-independent (Thompson, 2015).

### Pannexin-1 channels as conduits for release of the “gliotransmitter” ATP from type II cells

Panx-1 channels have pores large enough to allow passage of molecules <1.5 kDa in various cell types including astrocytes and central neurons (Dahl, 2015; Penuela et al., 2015; Thompson, 2015). These molecules include “gliotransmitters” such as ATP, GABA, and glutamate. Given the central role of ATP in CB neurotransmission (Nurse, 2014), it was of interest to determine whether the Panx-1 channels in type II cells acted as ATP release channels. To test this, P2XR-expressing petrosal neurons served as ATP biosensors in the CB co-cultures containing type 1/type II cell clusters (Zhang et al., 2012). In that study, selective stimulation of P2Y2R on type II cells with UTP led to depolarization and/or increased firing in several nearby petrosal neurons. These petrosal responses were reversibly inhibited by blockers of P2X2/3 receptors (PPADS) or Panx-1 channels (carbenoxolone) suggesting they arose from ATP released through Panx-1 channels (Zhang et al., 2012). Though petrosal neurons functioned as ATP biosensors in the latter study, the results led to the intriguing possibility that simply stimulating type II glial cells alone was sufficient to excite petrosal afferent terminals at the CB tripartite synapse, apparently without type I cell involvement. This occurred in spite of the unfavorably geometry present in this monolayer co-culture model, increasing the likelihood that it may also occur *in vivo*. If so, the proposed interaction could be facilitated by the close relation between type II cell processes and the afferent terminal (Platero-Luengo et al., 2014), as well as the possibility of physical coupling between P2X2/3R and Panx-1 channels as recently shown in co-immunoprecipitation studies (Li et al., 2017). This potential for type II cells alone to directly excite petrosal nerve endings via ATP release has further implications. For example, conditions associated with an increase in circulating levels of compounds such as angiotensin II, e.g., CIH-induced hypertension and CHF (Schultz et al., 2015), could lead to enhanced CB excitation solely via stimulation of AT_1_ receptors on type II cells. Additional independent support for the posit that type II cells can release ATP through Panx-1 channels was obtained in experiments using fura-2 Ca^2+^ imaging to test for possible crosstalk from type II to type I cells (Murali and Nurse, 2016). In that study carbenoxolone reversibly inhibited ATP-dependent crosstalk from type II to type I cells, as discussed further below. The ability of the spindle-shaped type II cells to release ATP, coupled with their expression of P2Y2R, also raise the possibility that intercellular Ca^2+^ waves may propagate within the network of interconnected type II cells and thereby facilitate delivery of ATP to the synaptic region.

### ATP-dependent crosstalk from type II to type I cells

The P2Y2R-[Ca^2+^]_i_-Panx-1 pathway discussed in the preceding sections implied a potential role for type II cells as ATP amplifiers due to the mechanism of “ATP-induced ATP release.” In addition to causing excitation at the sensory nerve terminal via P2X2/3R, ATP released through Panx-1 channels could in turn inhibit type I cells by negative feedback mechanisms involving P2Y1R or P2Y12R as discussed earlier. However, extracellular ATP at the CB chemosensory synapse can be metabolized by ectonucleotidases to adenosine (Conde and Monteiro, 2004; Holmes et al., 2017; Salman et al., 2017), which is excitatory at both the sensory nerve ending and type I cells (see above). This raised the question whether P2Y2R-mediated activation of Panx-1 channels in type II cells could lead to positive feedback stimulation of neighboring type I cells. Indeed, stimulation of type II cells with UTP often led to a delayed secondary rise in intracellular Ca^2+^ in nearby type I cells (Murali and Nurse, 2016). Consistent with a role for adenosine, generated from ATP catabolism after release through Panx-1 channels, the delayed type II cell Ca^2+^ response was strongly inhibited by the Panx-1 blocker carbenoxolone, the adenosine A2aR blocker SCH58261, and the ecto-5′-nucleotidase blocker AOPCP (Murali and Nurse, 2016). Thus, type II cells may participate in the integrated sensory response of CB by contributing to both ATP and adenosine synaptic pools.

## Plasticity in CB neurotransmitter functions during exposure to chronic hypoxia

A prominent feature of the CB is its remarkable plasticity following exposure of animals to different patterns of hypoxia including chronic sustained and chronic intermittent hypoxia or CIH (Kumar and Prabhakar, 2012). The reader is referred to other excellent reviews for a discussion of CB plasticity following CIH (Prabhakar et al., 2015; Iturriaga, 2017). Changes in ion channel expression leading to increased membrane excitability of type I cells, as well as alterations in a variety of neurotransmitter systems, are well-described CB plasticity mechanisms that occur during exposures to chronic sustained hypoxia (Prabhakar and Jacono, 2005; Powell, 2007). We focus here mainly on purinergic neurotransmitter mechanisms underlying CB plasticity during chronic hypoxia, as may occur during ascent to altitude. In the latter condition, an adaptive process known as ventilatory acclimatization to hypoxia (VAH) ensures an increase in sensitivity of the CB chemoreflex such that for a given PO_2_ there is an augmented CSN discharge frequency over and above that present before acclimatization (Prabhakar and Jacono, 2005; Powell, 2007; Kumar and Prabhakar, 2012). Given the central role of ATP and adenosine in CB chemoexcitation as previously discussed, perhaps it is not surprising that alterations in purinergic signaling pathways have been implicated in VAH. For example, when adult rats were reared under chronic hypoxia (12% O_2_) for 7 days, infusion of the non-specific adenosine receptor antagonist 8-sulpho-phenyltheophylline (8-SPT) attenuated the increase in respiratory frequency evoked by acute hypoxia (Walsh and Marshall, 2006). Similarly when caffeine, a non-specific adenosine receptor antagonist, was included in the drinking water of chronically-hypoxic rats the CB sensory output was markedly inhibited (Conde et al., 2012b). These data point to a major contribution of adenosine signaling to the mechanisms underlying VAH.

Because surface-located ectonucleotidases control adenosine levels at the CB tripartite synapse (see above), it was of interest to determine whether expression of these enzymes was regulated by chronic hypoxia as occurs in other cell types, e.g., smooth muscle cells (Koszalka et al., 2004; Robson et al., 2005). In a recent study, exposure of rats to chronic hypobaric hypoxia (~60 kPa, simulating an altitude of ~4,300 m) for 5–7 days led to an ~2x increase in expression of NTPDase3 and ecto-5′-nucleotidase/CD73 mRNAs, concomitant with a significant decrease in expression of NTPDase1 and NTPDase2 mRNAs (Salman et al., 2017). Assuming changes in mRNA expression correlate with parallel changes in protein expression, the modifications seen in chronic hypoxia favor a shift toward adenosine signaling in the CB because NTPDase3 efficiently hydrolyses ATP to AMP, whereas ecto-5′-nucleotidase catalyzes the conversion of AMP to adenosine in the rate-limiting step (Zimmermann, 2000). This shift could be further aided by the rapid depletion of the ATP and ADP pools because both purines are natural physiological inhibitors of ecto-5′-nucleotidase (Lecka et al., 2010). Further, the tendency of ecto-5′nucleotidase and adenosine A2aR to co-localize, as previously demonstrated in the striatum (Augusto et al., 2013), could enable the efficiency of adenosine-A2aR interactions at the membranes of both type I cells and sensory nerve terminals. The proposed depletion of extracellular ATP and ADP pools during chronic hypoxia could facilitate type I cell depolarization and increased sensitivity by diminishing their negative feedback influences mediated by interactions with P2Y1R and/or P2Y12R on type I cells (Xu et al., 2006; Carroll et al., 2012). Evidence for an increased role for adenosine signaling during VAH is further supported by the observation that acute hypoxia-evoked adenosine release is markedly potentiated in isolated whole CBs from chronically hypoxic rats (Conde et al., 2012b). It is also possible that autoregulatory “push-pull” mechanisms could prevent adenosine-mediated overexcitation. For example, high levels of adenosine in the micromolar range could activate low affinity A2bR on type I cells and facilitate secretion of dopamine (Conde et al., 2008; Livermore and Nurse, 2013), which inhibits CB function via pre- and postsynaptic D2R on type I cells and petrosal nerve endings (see earlier discussion). Indeed, an increase in both basal and hypoxia-evoked dopamine release has been reported for chronically hypoxic CB chemoreceptor cells (Jackson and Nurse, 1997; Conde et al., 2012b). Taken together, these studies support a major shift toward adenosine-A2R signaling in the CB after exposure to chronic hypoxia *in vivo*.

## Conclusions and future directions

**Figure 6:**
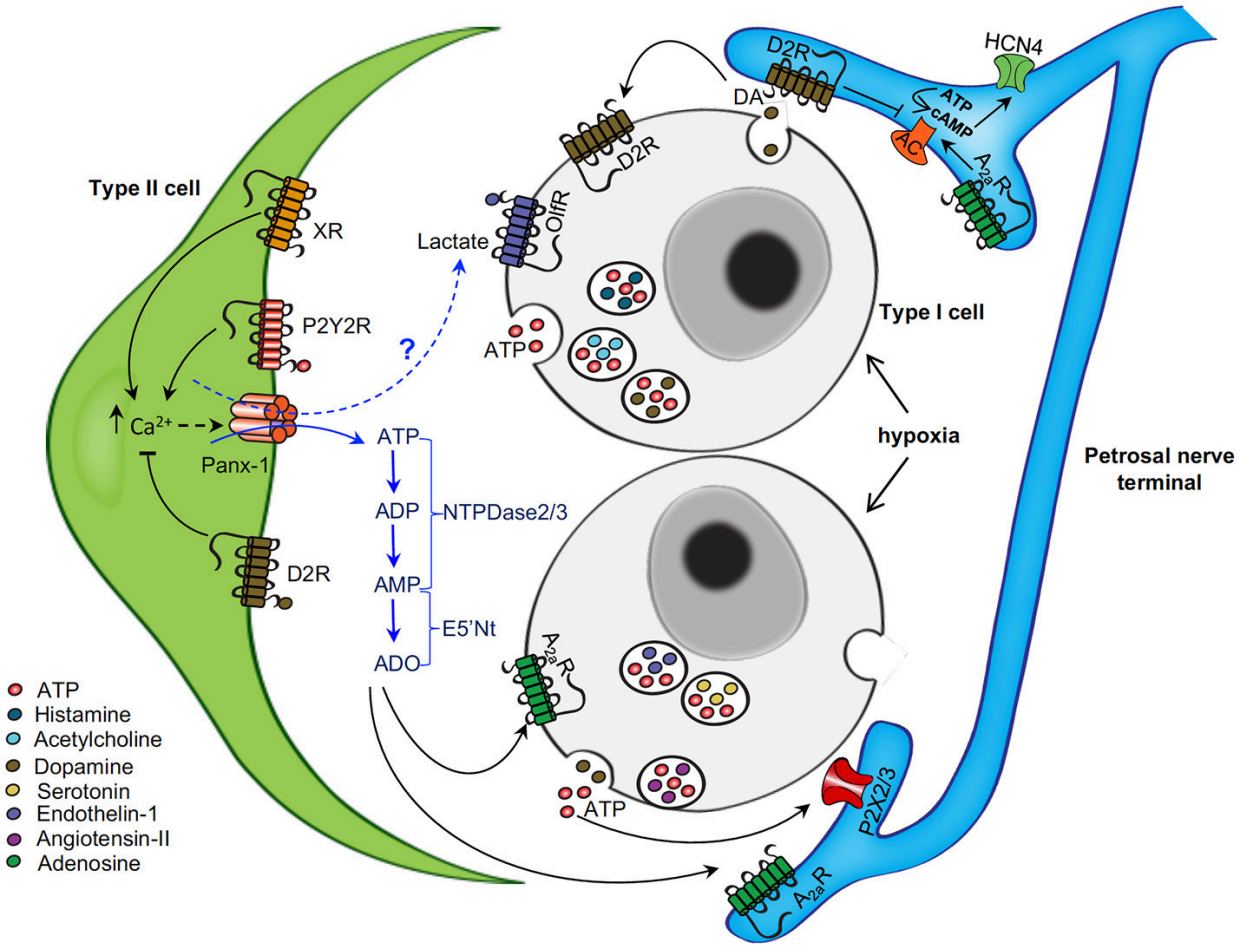
Schematic representation of neurotransmitter functions at the tripartite synapse of the rat carotid body. The diagram shows some of the neurotransmitter mechanisms that are likely to operate at the carotid body sensory synapse during acute hypoxia. Chemoreceptor type I cells depolarize and release ATP, dopamine (DA), and other neurochemicals (X) during hypoxia. ATP acts postsynaptically on ionotropic P2X2/3R on petrosal nerve terminals causing excitation. ATP also activates P2Y2R on adjacent glial-like type II cells, leading to a rise in intracellular Ca^2+^ and activation of pannexin-1 (Panx-1) channels which act as conduits for the further release of ATP; Panx-1 channels may also release other “gliotransmitters” such as lactate, a potential ligand for Olf78 receptors present on type I cells. Extracellular ATP is degraded by a series of surface-located nucleotidases, including NTPDase2,3 and ecto-5-nucleotidase, to adenosine (ADO). ADO could then act in a paracrine manner to activate A2aR on nearby type I cells (causing inhibition of TASK channels) and sensory nerve terminals (causing modulation HCN4-containing *I*_h_ channels), leading to increased excitation. DA released during hypoxia may act on inhibitory D2R present on adjacent type I cells, and possibly type II cells, so as to inhibit intracellular Ca^2+^ signaling. Also, DA may act postsynaptically on the sensory nerve terminal to inhibit the HCN channels and action potential frequency. Other released neurochemicals (X), including angiotensin II, 5-HT, ACh, and ET-1, may act on corresponding G-protein coupled receptors (XR) on type II (and likely type I) cells to increase intracellular Ca^2+^ signaling and activate Panx-1 channels. The action of some of these neurochemicals may become more relevant in pathophysiological conditions (e.g., after chronic or intermittent hypoxia) when carotid body sensitivity is augmented. Omitted for clarity are: (i) negative feedback inhibition of type I cell function by ATP and ADP acting via P2Y1 or P2Y12 receptors; (ii) stimulatory effect of low affinity A2b receptors on DA secretion from type II cells; and (iii) inhibitory effects of GABA on presynaptic GABA(B) receptors on type I cells and on postsynaptic GABA(A) receptors on the sensory nerve terminal. Figure adapted from Murali et al. (2017).

In this review we considered the evidence that the integrated CB output is determined largely by neurochemical interactions at the tripartite synapse formed by type I chemoreceptor cells, type II glial cells, and sensory nerve endings. Several of these interactions involving fast-acting ionotropic receptors and slower-acting G-protein coupled receptors are summarized in Figure 6. There is a growing consensus that the purines ATP and adenosine are key players in mediating CB chemoexcitation and their actions involve P2 and P1 receptors located at pre- and postsynaptic sites. While the evidence in most species has long favored an inhibitory role for dopamine acting via D2R on type I cells, we considered novel data showing that dopamine-D2R signaling might also inhibit the sensory afferent discharge via modulation of the *I*_h_ current that controls action potential frequency. Moreover, recent evidence suggests that the excitatory effects of P2Y2R-mediated Ca^2+^ signaling in varying subpopulations of type II cells might be inhibited by dopamine (Leonard and Nurse, 2017), or histamine (Nurse et al., 2018). The possibility that other CB neurochemicals including small molecules (e.g., histamine and 5-HT) and neuropeptides (e.g., angiotensin II) might act postsynaptically on *I*_h_ so as to regulate firing frequency requires future investigation. We also considered several G-protein coupled pathways by which type II glial cells might contribute to synapse integration; all of these converge on the Ca^2+^-dependent activation of Panx-1 channels which act as conduits for ATP release. These channels are known to release other “gliotransmitters” in the CNS, raising the possibility that type II cells may also release other neuroactive chemicals to further shape or fine-tune the CB sensory output. For example, open Panx-1 channels in CNS astrocytes may stimulate release of lactate which serves as an extracellular energy substrate as well as a signaling molecule for glia-neuron communication (Karagiannis et al., 2016). In this regard, the lactate receptor Olfr78 that is highly expressed in type I cells has been proposed as a potential “hypoxia sensor” (Chang, 2017), raising the possibility that this pathway could be activated during acute hypoxia by lactate release from type II cells via Panx-1 channels (see Figure 6). Finally, this review considered some of the purinergic mechanisms that are likely to contribute to the increased sensitivity of the CB during exposure to chronic hypoxia. Whether similar or overlapping changes occur during pathophysiological conditions that lead to increased CB chemosensitivity, e.g., exposure to intermittent hypoxia (Kumar and Prabhakar, 2012), requires future investigation.

## Author contributions

EL performed experiments, analyzed data, helped prepare figures, reviewed literature and contributed to first draft. SS performed experiments, analyzed data, helped prepare figures, reviewed literature and contributed to first draft. CN organized review subheadings, helped interpret data, helped prepare figures, reviewed literature and contributed to first draft.

### Conflict of interest statement

The authors declare that the research was conducted in the absence of any commercial or financial relationships that could be construed as a potential conflict of interest.
